# Mastication‐Induced Electrical Stimulation Activates Prg4^+^ Chondroprogenitors for Osteoarthritis Therapy

**DOI:** 10.1002/advs.75725

**Published:** 2026-05-19

**Authors:** Shi‐Yang Feng, Jing‐Rong Cheng, Yu Qin, Jie Lei, Chen‐Chen Gao, Hong‐Ying Fu, Li‐Ping Wu, Thanh D. Nguyen, Xu‐Liang Deng, Kai‐Yuan Fu, Yang Liu

**Affiliations:** ^1^ Center For TMD and Orofacial Pain Peking University School and Hospital of Stomatology Beijing China; ^2^ NMPA Center for Innovation and Research in Regulatory Science and Beijing Key Laboratory of Digital Stomatology National Engineering Research Center of Oral Biomaterials and Digital Medical Devices Beijing China; ^3^ National Center for Stomatology and National Clinical Research Center for Oral Diseases and NHC Research Center of Engineering and Technology for Computerized Dentistry Beijing China; ^4^ Beijing Key Laboratory of Biomaterials for Oral Disease Peking University School and Hospital of Stomatology Beijing China; ^5^ Department of Dental Materials and Dental Medical Devices Testing Center Peking University School and Hospital of Stomatology Beijing China; ^6^ Department of Orthopedics Peking University Third Hospital Beijing China; ^7^ Department of Biomedical Engineering University of Connecticut Storrs Connecticut USA; ^8^ Institute of Materials Science Polymer Program University of Connecticut Storrs Connecticut USA; ^9^ Department of Mechanical Engineering University of Connecticut Storrs Connecticut USA

**Keywords:** cartilage regeneration, osteoarthritis, piezoelectrical stimulation, single‐cell RNA‐sequencing

## Abstract

Osteoarthritis (OA) is a whole‐joint degenerative disease primarily driven by excessive mechanical overloading, yet current therapies fail to harness biomechanical cues to halt cartilage degeneration. Here, we developed mastication‐driven magnesium‐incorporated piezoelectric nanofibers (MagPie) that could convert joint loading into localized electrical stimulation while releasing Mg^2^^+^ to neutralize the acidic microenvironment. Under mechanical stimulation, MagPie promoted chondrocyte anabolism and suppressed inflammation in vitro. In a rat temporomandibular joint OA model induced by overloading, MagPie, coupled with physiological mastication, restored the damaged osteochondral unit. Through single‐cell transcriptomics, an electro‐sensitive Prg4^+^ chondroprogenitor‐like cell population was identified as a key mediator of cartilage regeneration. Mechanistically, MagPie‐activated Prg4^+^ cells enhanced anabolic activity via the ECM‐mediated FAK‐PI3K‐Akt axis and ameliorated the arthritic microenvironment by shifting macrophage polarization toward an anti‐inflammatory phenotype. Genetic ablation of *Prg4* attenuated the therapeutic efficacy of MagPie. Together, these findings uncover a mechanism of electrosensitive tissue repair and establish a ‘trash‐to‐treasure’ strategy that harnesses joint overloading to activate endogenous regenerative programs for OA therapy.

## Graphical abstract

This study presents a ″trash‐to‐treasure″ strategy that converts harmful joint overloads into beneficial electrical cues. The biodegradable MagPie, by providing piezoelectrical stimulation and releasing Mg^2^^+^ during mastication, exerts synergistic pro‐regenerative and anti‐inflammatory effects on Prg4^+^ cells via the ECM‐mediated FAK‐PI3K‐Akt axis, thereby ameliorating the arthritic microenvironment and facilitating cartilage repair.

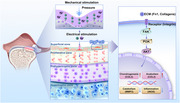

## 1 Introduction

Osteoarthritis (OA) is a highly prevalent degenerative disease that affects the entire joint [1]. As a leading cause of pain and physical disability, OA imposes a substantial and increasing health burden with notable implications for affected individuals, healthcare systems, and socioeconomic costs [2, 3]. The temporomandibular joint (TMJ), a unique craniofacial joint essential for normal masticatory functions, is one of the most frequently affected sites of OA [4]. TMJOA exhibits a prevalence of 9.8% in the general population, accounts for an estimated annual healthcare cost of $4 billion, and represents the leading cause of non‐dental chronic pain in the orofacial region [5]. Diverging from typical age‐related OA observed in weight‐bearing joints, TMJOA predominantly affects individuals aged from 20 to 40 due to the high incidence of anterior disc displacement (ADD) in younger populations [6, 7]. As TMJOA progresses, patients often develop condylar structural abnormalities, leading to dentofacial deformities, occlusal derangements, and functional disabilities, which collectively compromise quality of life [8].

TMJ condylar cartilage serves as the growth center of the mandibular skeletal system, regulating jaw morphogenesis and development [9]. Distinct from most other joints, the TMJ surface is covered by fibrocartilage rather than hyaline cartilage [10]. The stem/progenitor cell pool within fibrocartilage can differentiate into mature chondrocytes, enabling the condyle to undergo active remodeling throughout its lifetime and respond rapidly to mechanical loading [11, 12, 13, 14]. However, excessive loading induced by ADD leads to depletion of this progenitor pool, drives degenerative cartilage degeneration, and ultimately impairs mandibular growth and function [15, 16, 17]. Due to the restricted regenerative capacity of cartilage, current therapeutic strategies primarily focus on symptom relief rather than structural restoration. Consequently, patients with advanced‐stage TMJOA often require surgical joint replacement [18]. Although recent clinical trials have explored intra‐articular delivery of exogenous stem/progenitor cells, such as adipose‐derived stem cells and nasal septum‐derived chondroprogenitors, challenges, including immune rejection and complex preparation processes, remain [19, 20, 21]. Therefore, it is imperative to explore approaches that can activate and sustain the endogenous fibrocartilage stem/progenitor cell pool to promote cartilage regeneration and preserve condylar integrity. Recent evidence suggested that Procr^+^ chondroprogenitors, a subpopulation of Prg4^+^ cells, are capable of sensing mechanical stimuli, highlighting the potential for selectively targeting mechanosensitive cell populations to drive cartilage regeneration [22].

As the typical piezoelectric tissue, cartilage is highly responsive to electrical stimulation (ES) and functionally coupled with subchondral bone in maintaining joint homeostasis [23]. Accumulating evidence has demonstrated the beneficial effects of ES on cellular activity and tissue regeneration [24, 25, 26, 27]. Various ES‐based strategies have been explored to enhance extracellular matrix (ECM) synthesis and promote chondrogenic differentiation [26, 27]. Among these, piezoelectric materials, which are capable of converting mechanical stimuli into an electrical signal in a non‐invasive manner, have shown particular promise for cartilage repair and bioelectronic therapies [28]. Our previous study has reported that a biodegradable piezoelectric poly (L‐lactic acid) (PLLA) scaffold could function as a battery‐free electrical stimulator to promote hyaline cartilage regeneration in a rabbit model of knee osteochondral defects [29]. However, its effectiveness in other joint environments remains unclear. Additionally, the key cell populations and mechanisms involved in piezoelectrically induced cartilage repair have yet to be fully elucidated.

In this study, we hypothesized that a piezoelectric film could harness joint overloading to generate electrical cues and release Mg^2+^, which may be beneficial for cartilage regeneration and inflammation suppression. We develop mastication‐driven magnesium‐incorporated piezoelectric nanofibers (MagPie) capable of releasing Mg^2+^, neutralizing acidic pH, and providing electrical stimulation. We envision that if repetitive joint loading acts on the MagPie during mastication, more charges and Mg^2^^+^ would be released on the condylar surface, thus further promoting cartilage repair and improving the inflammatory microenvironment in the TMJ. To test this hypothesis, we attempted to: (i) evaluate the ability of MagPie to harness ADD‐induced mechanical overloading for enhancing cartilage regeneration in vitro and in vivo, and (ii) identify the key responsive cell populations and underlying mechanisms involved in TMJOA electrotherapy using single‐cell RNA sequencing.

## 2 Results

### 2.1 PLLA Films Alone Fail to Promote Osteochondral Repair in TMJOA

**FIGURE 1:**
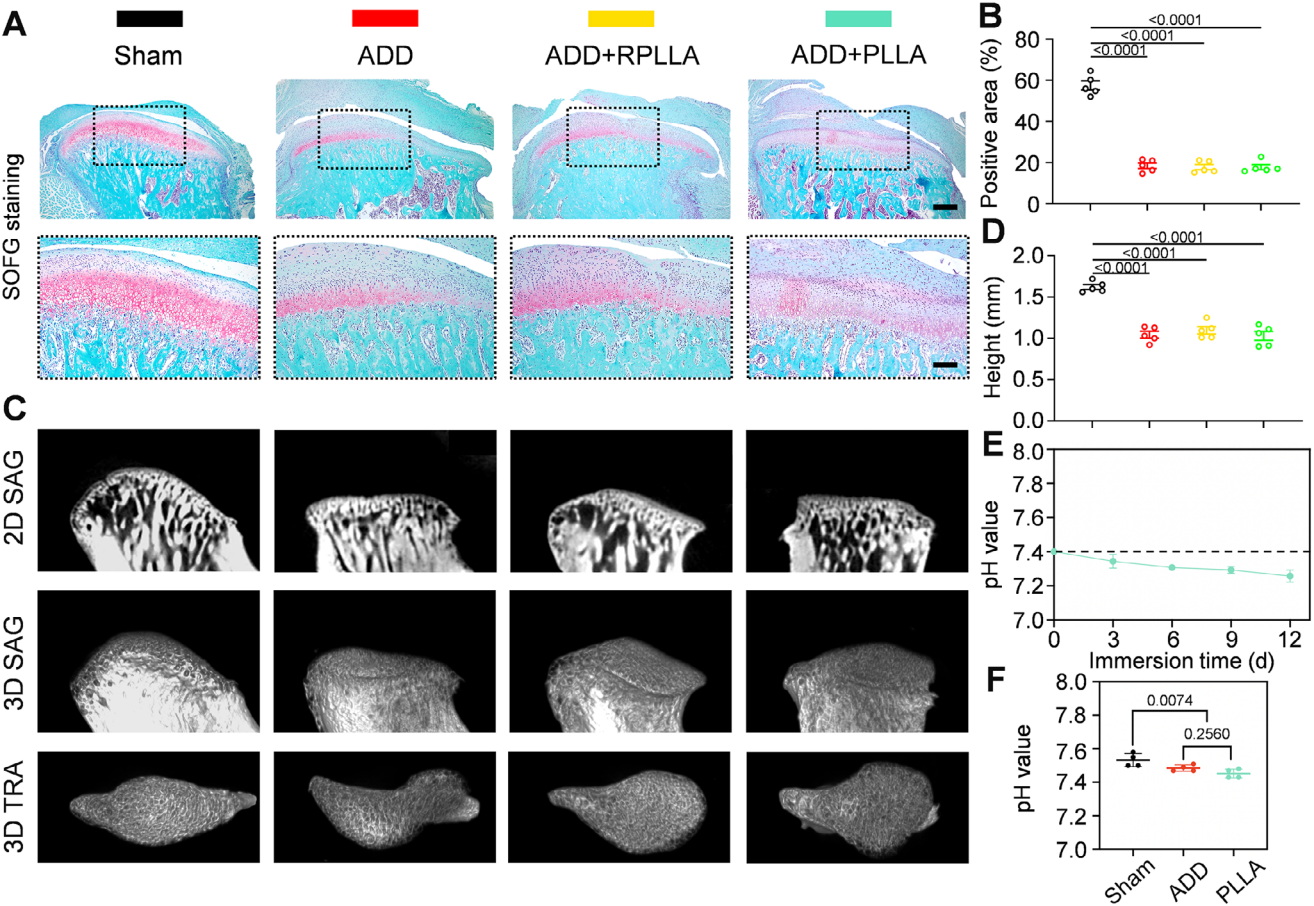
PLLA films alone fail to promote osteochondral repair in TMJOA. (A) Representative images of SOFG staining in TMJ sagittal sections. Scale bar: (top row) 500 µm, (bottom row) 200 µm. (B) Quantitative analysis of the percentage of red area in cartilage. Data were presented as means and SEM. *n* = 5 per group. (C) Micro‐CT images of TMJ condyles in 2D sagittal view, 3D sagittal view, 3D transverse view. Scale bar: 1 mm. (D) Quantitative analysis of height of TMJ condylar heads. Data were presented as means and SEM. *n* = 5 per group. (E) pH value of PLLA immersed in PBS for 12 days. *n* = 3 per group. (F) pH value of TMJ synovial fluid before and after PLLA implantation *n* = 3 per group.

Piezoelectric PLLA films were synthesized as previously reported [29]. To assess their therapeutic potential in ADD‐induced TMJOA, PLLA film was implanted onto the condylar surface, and pathological changes in the osteochondral unit were observed. Safranin O‐fast green (SOFG) staining revealed significant proteoglycan loss in the condylar cartilage matrix at 4 weeks after ADD surgery. Micro‐computed tomography (CT) further demonstrated condylar flattening and subchondral bone sclerosis at 4 weeks post‐ADD surgery. Neither nonpiezoelectric PLLA with random orientation (Random PLLA, RPLLA) nor piezoelectric PLLA film implantation attenuated these degenerative changes in both condylar cartilage and subchondral bone (Figure 1A–D). These findings contrast with previous reports in the knee joint, where PLLA films under mechanical loading enhanced cartilage regeneration in a rabbit osteochondral defect model [29]. To explore the reason for the lack of therapeutic efficacy of PLLA films in the ADD‐induced TMJOA model, we examined pH changes with PLLA degradation in vitro and in vivo. The pH value of PLLA immersed in phosphate buffer saline (PBS) decreased gradually (Figure 1E). We further collected synovial fluid from rats and found a lower intra‐articular pH value in the PLLA implantation group, indicating the formation of an acidic microenvironment, which may result from both PLLA degradation and the associated inflammatory response. (Figure 1F).

### 2.2 Characteristics of the Magnesium Incorporated Piezoelectric Nanofibers (MagPie)

**FIGURE 2:**
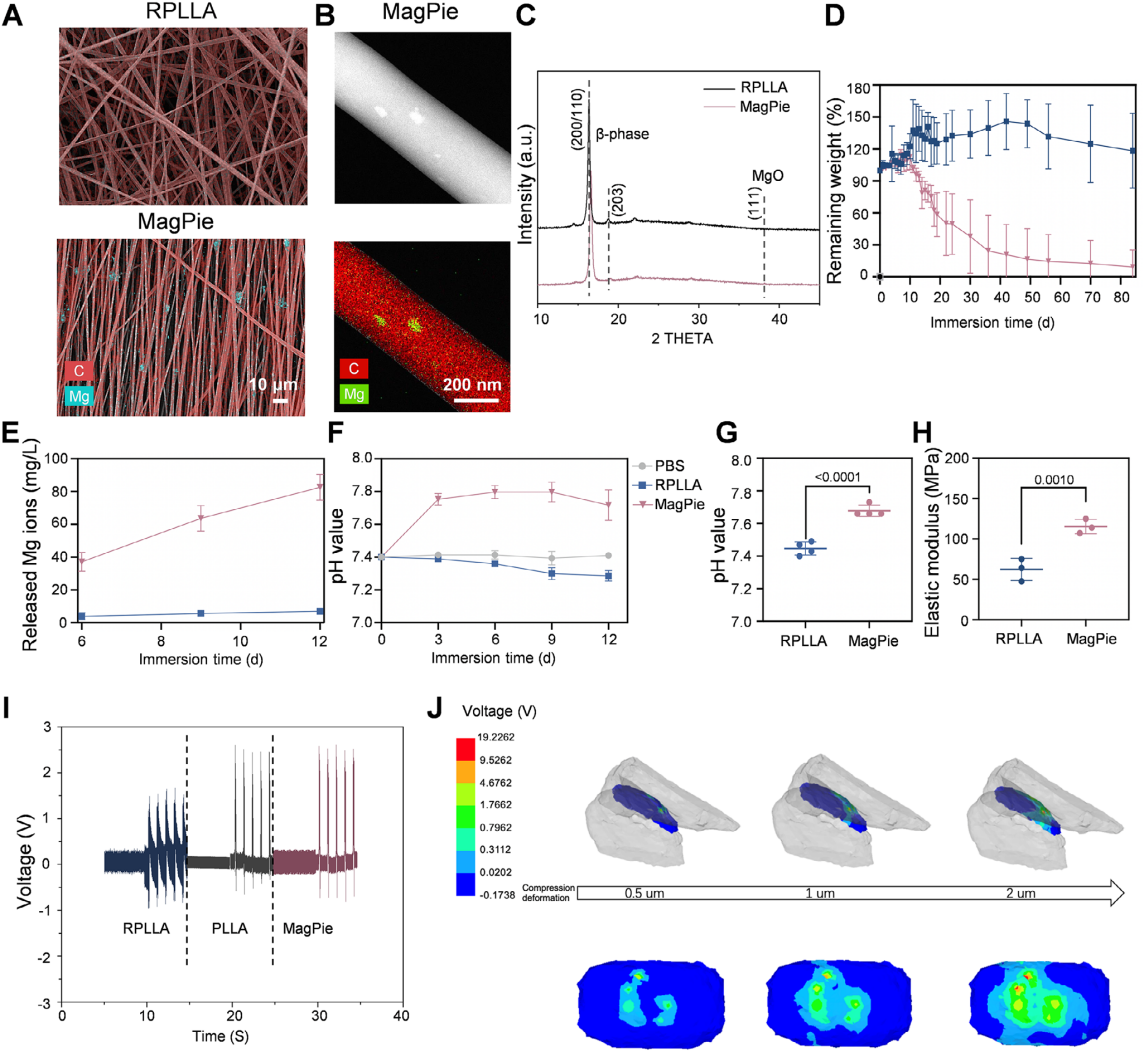
Characterizations of the MagPie. (A) SEM and EDS images of RPLLA and MagPie. Carbon was presented in red, and Mg^2+^ was presented in blue. Scale bar: 10 µm. (B) TEM images of MagPie. Carbon was presented in red, and Mg^2+^ was presented in green. Scale bar: 200 nm. (C) XRD spectra of RPLLA and MagPie. (D) The degradation performance of RPLLA and MagPie in PBS. *n* = 3 per group. (E) Mg^2+^ releasing amount of RPLLA and MagPie in PBS. *n* = 3 per group. (F) pH changes of RPLLA and MagPie in PBS. *n* = 3 per group. (G) pH changes of synovial fluid in TMJOA rats with RPLLA and MagPie. *n* = 4 per group. (H) Elastic modulus of RPLLA and MagPie. Data were expressed as individual data points and the mean value. *n* = 3 per group. (I) Output voltage response in the process of films applying force. (J) The simulation of the film motion process in vivo and the voltage distribution.

To overcome the limitations of PLLA and achieve enhanced TMJOA cartilage repair, we developed magnesium‐incorporated piezoelectric nanofibers (MagPie). It is generally recognized that the orientation of fibers is a key determinant of piezoelectricity. RPLLA with random orientation and MagPie with aligned fiber orientation were shown in Figure 2A. RPLLA films were used as a control. The distribution of magnesium oxide (MgO) in the MagPie fiber was observed (Figure 2B). X‐ray diffraction (XRD) analysis confirmed the crystalline structures of β‐form PLLA and MgO in MagPie, peaks at PLLA 200/110 and 203 planes, and at MgO 111 plane (Figure 2C). Incorporation of MgO accelerated degradation (Figure 2D) and resulted in a maximum daily Mg^2+^ releasing rate of 8.8 ± 0.2 mg/L (Figure 2E). Moreover, MagPie rapidly stabilized the pH at approximately 7.7 ± 0.1 (Figure 2F). The pH value of synovial fluid further confirmed that MagPie established a favorable microenvironment for tissue homeostasis (Figure 2G). Tensile test demonstrated that MagPie exhibited a higher elastic modulus along the fiber direction (Figure 2H). Piezoelectric effects of the films were evaluated under a 1 Hz strike, the results showed that the output voltage of piezoelectric PLLA, MagPie were higher than nonpiezoelectric RPLLA, indicated that MgO incorporation did not compromise piezoelectric performance (Figure 2I). Previous studies reported that the mean pressure at the anterior condylar surface reached 931.72 ± 56.50 kPa under disc displacement, compared with 184.68 ± 54.60 kPa under normal conditions, as measured in vivo. Meanwhile, Liu et al. established the pressure‐voltage relationship based on the intrinsic piezoelectric properties of PLLA [29, 30]. Integrating these parameters, finite element simulations revealed that MagPie generated the highest output voltage at the anterior condylar surface in the rat ADD‐induced TMJOA model (Figure 2J), corresponding to the primary contact region during joint movement. Therefore, we implanted films on the condylar anterior surface in subsequent animal experiments to obtain the maximum voltage.

### 2.3 MagPie Promotes Chondrocyte Anabolism and Attenuates Catabolism in Vitro

**FIGURE 3:**
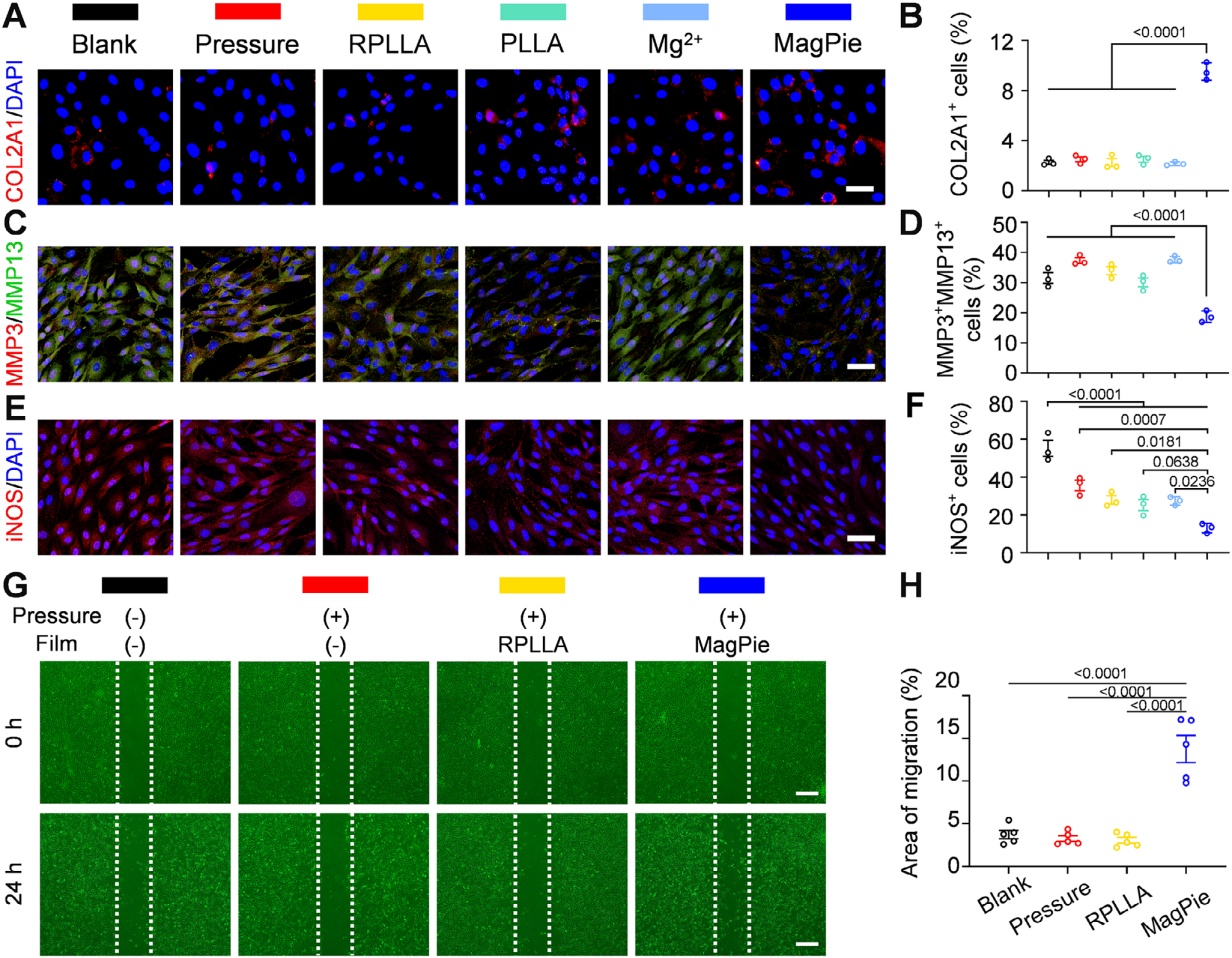
MagPie exerts growth‐promoted effects on chondrocytes in vitro. (A) Immunofluorescence staining of COL2A1 in chondrocytes cultured under different conditions. Scale bar: 50 µm. (B) Quantitative analysis of the percentage of COL2A1‐positive chondrocytes. Data were presented as means and SEM. *n* = 3 per group. (C) Immunofluorescence staining of MMP3 (red) and MMP13 (green) in chondrocytes cultured under different conditions. Scale bar: 50 µm. (D) Quantitative analysis of the percentage of MMP3 & MMP13 double‐positive chondrocytes. Data were presented as means and SEM. *n* = 3 per group. (E) Immunofluorescence staining of iNOS in chondrocytes cultured under different conditions. Scale bar: 50 µm. (F) Quantitative analysis of the percentage of iNOS positive chondrocytes. Data were presented as means and SEM. *n* = 3 per group. (G,H) Representative images (G) and quantitative analysis (H) of chondrocyte migration. Scale bar: 200 µm. Data were presented as means and SEM. *n* = 5 per group.

To evaluate the influence of MagPie on chondrocytes, primary chondrocytes isolated from rat condyles were co‐cultured with biodegradable films and subjected to cyclic mechanical loading. Six experimental conditions were established: (1) chondrocytes cultured in complete medium without loading; (2) chondrocytes exposed to cyclic loading alone; (3) chondrocytes co‐cultured with RPLLA film under cyclic loading; (4) chondrocytes cocultured with PLLA film under cyclic loading; (5) chondrocytes cocultured with Mg^2+^ medium under cyclic loading; (6) chondrocytes cocultured with MagPie under cyclic loading. Chondrocyte metabolic activity was assessed by the expression of chondrogenic marker COL2A1 and matrix metalloproteinases (MMPs), MMP3 and MMP13. Among all groups, MagPie under cyclic loading most effectively promoted extracellular matrix synthesis while suppressing cartilage degradation (Figure 3A–D), suggesting a synergistic effect of piezoelectric charges and Mg^2^^+^ on chondrogenesis. We next examined the inflammatory response of chondrocytes following IL‐1β stimulation under each intervention. Notably, chondrocytes in the MagPie group exhibited markedly reduced expression of inducible nitric oxide synthase (iNOS) compared with other groups (Figure 3E,F).

To further explore the effect of MagPie on cell migration, a scratch assay was conducted to illustrate cell distribution in different groups at the time of wound creation and 24 h later (Figure 3G). Clearly, the MagPie group exhibited the best outcome of chondrocyte migration (Figure 3H). In addition, the biocompatibility of the biodegradable films was evaluated. These films did not show any side effects on cell viability via Live/Dead staining and CCK‐8 assay (Figure S1).

### 2.4 MagPie Preserves Joint Structure and Delays TMJOA Progression

**FIGURE 4:**
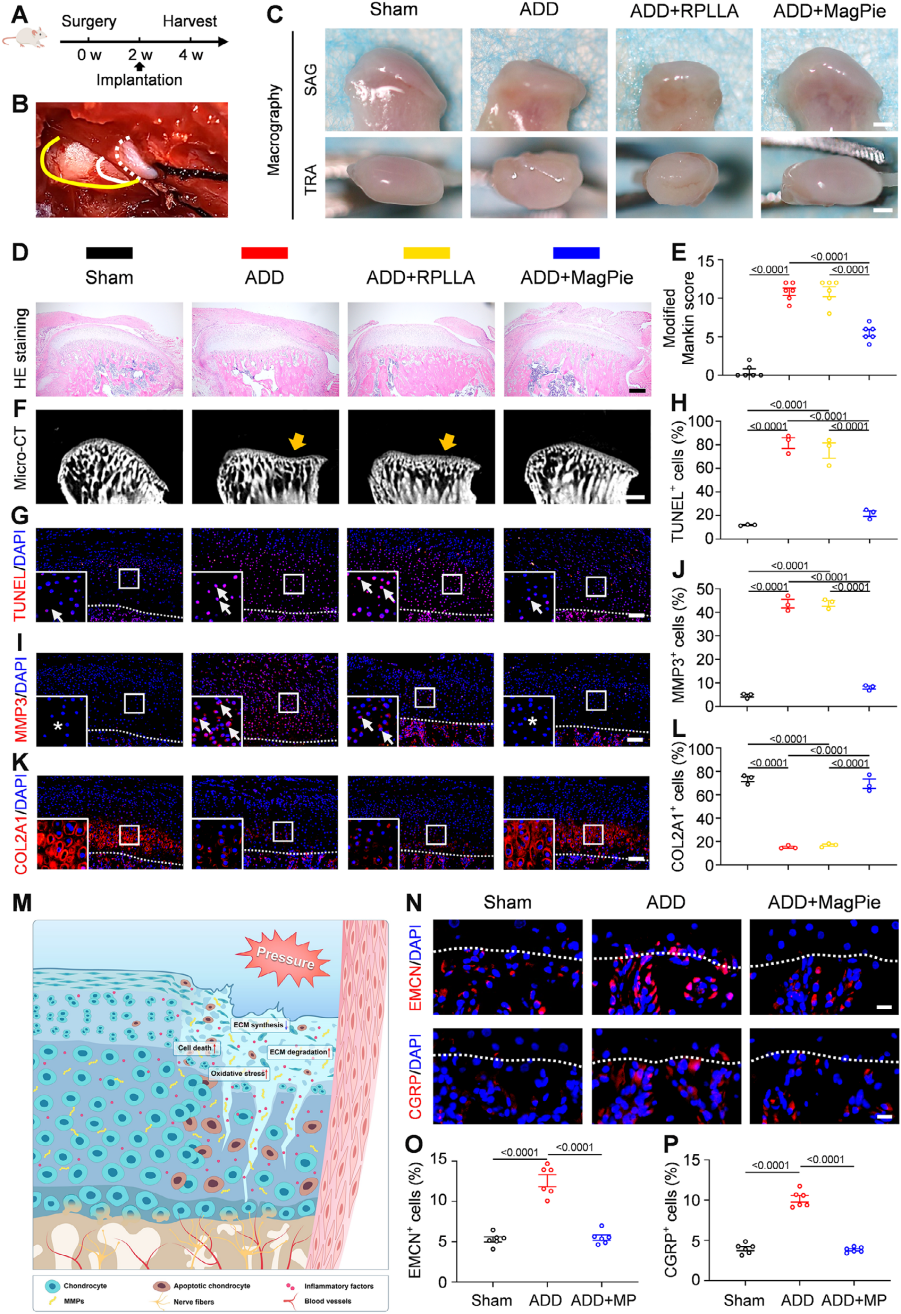
MagPie improves condylar cartilage and subchondral bone in an ADD‐induced TMJOA rat model. (A) Schematic diagram of the animal experiment operation protocol. (B) The relative position of displaced disc, implanted film, and condyle. The white dotted line shows the margin of the displaced disc. The white solid line shows margin of the implanted film. The yellow solid line shows the contour of condyle. (C) Macrography of TMJ condyles. Scale bar: 1 mm. (D) Representative images of HE staining. Scale bar: (top row) 500 µm, (bottom row) 200 µm. (E) Quantitative analysis of the modified Mankin score. Data were presented as means and SEM. *n* = 6 per group. (F) Micro‐CT images of TMJ condyles. The yellow arrows indicate condylar surface flattening and local sclerosis. Scale bar: 1 mm. (G,H) Representative images and quantitative analysis of TUNEL positive cells. The white arrows indicate positive cells in cartilage. Scale bar: 100 µm. Data were presented as means and SEM. *n* = 3 per group. (I,J) Representative images and quantitative analysis of MMP3 positive cells. The white arrows indicate positive cells, and the asterisks indicate negative signals in cartilage. Scale bar: 100 µm. Data were presented as means and SEM. *n* = 3 per group. (K,L) Representative images and quantitative analysis of COL2A1 positive cells. Scale bar: 100 µm. *n* = 3 per group. (M) Schematic illustration depicting the microstructural features of condylar cartilage and subchondral bone under pressure. (N) Representative images showing neurovascular formation. The white dotted line represents the demarcation between condylar cartilage and subchondral bone. Scale bar: 20 µm. (O,P) Quantitative analysis of the percentage of EMCN positive cells and CGRP positive cells in osteochondral interface. Data were presented as means and SEM. *n* = 6 per group.

Building on the favorable in vitro performance of MagPie on chondrocyte anabolism and inflammation, we next evaluated its therapeutic efficacy in vivo. A rat TMJOA model was established via surgical induction of ADD. Two weeks after ADD induction, joints exhibited early degeneration, characterized by reduced cartilage proteoglycan content and increased unmineralized bone. By four weeks post‐surgery, degenerative changes, including cartilage degeneration and subchondral bone sclerosis, became evident, which underscored the progression of TMJOA [30, 31]. Based on these observations, biodegradable films were then implanted onto the anterior condylar surface at 2 weeks, and TMJs were harvested at 4 weeks after surgery (Figure 4A,B).

To determine whether implantation altered joint loading, masticatory activity was assessed by monitoring food intake. No significant difference was observed among treatment groups (Figure S2), indicating that the implanted films did not interfere with mastication and associated mechanical loading. Remarkably, in the ADD‐induced TMJOA model, MagPie treatment markedly restored cartilage and subchondral bone structure at 4 weeks after surgery (Figure 4C–F). Immunofluorescence staining corroborated these findings, manifesting reduced apoptosis chondrocytes, decreased MMP3‐positive cells, and increased COL2A1 expression in MagPie‐treated joints (Figure 4G–L). These data suggest that, under physiological mastication, MagPie harnesses pathological mechanical overloading to promote condylar cartilage regeneration.

Given that endochondral angiogenesis and neurogenesis contribute to TMJOA progression (Figure 4M) [32, 33], we next assessed vascular and neural changes. In the ADD‐induced TMJOA model, we found an increased distribution of blood vessels and nerve fibers in subchondral bone, with infiltration into the osteochondral interface of condyle. Conversely, MagPie treatment markedly reduced vascular and neural ingrowth at the osteochondral junction (Figure 4N–P). Consistently, MagPie alleviated TMJOA‐associated osseous alterations, including condylar flattening, subchondral bone sclerosis, and osteophyte formation, at 4 weeks after ADD surgery (Figure S3A). Micro‐CT analysis revealed that MagPie implantation significantly improved condylar morphology, as evidenced by increased condylar head height and restoration of bone mineral density (BMD) to normative values (Figure S3B,C). These data indicate that MagPie, by converting joint overloading into localized electrical stimulation and Mg^2^^+^ release, improve subchondral bone microstructure and attenuate TMJOA progression.

### 2.5 MagPie Activates Prg4^+^ Cells to Promote Condylar Cartilage Repair

**FIGURE 5:**
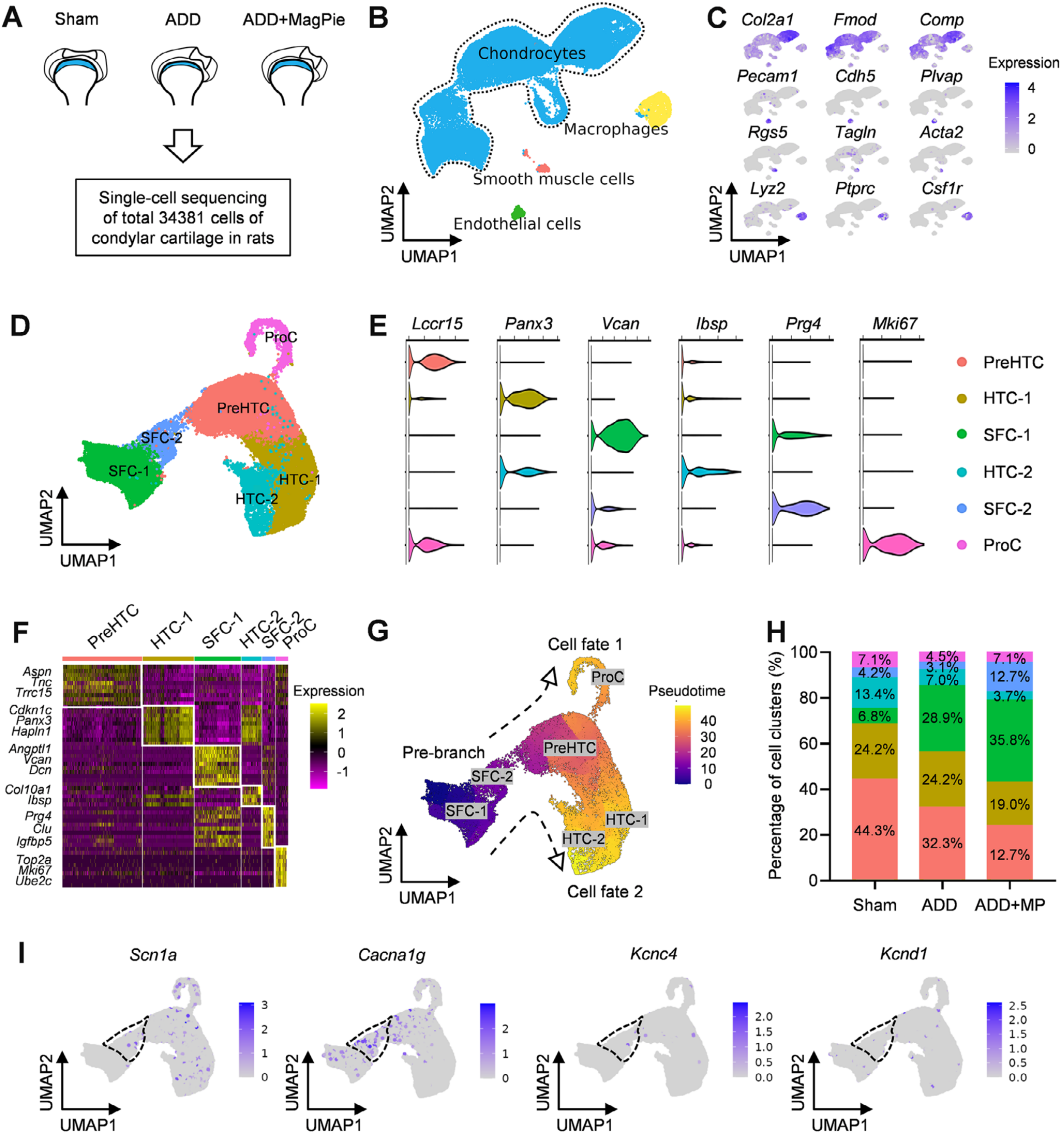
Single‐cell RNA sequencing identifies Prg4^+^ cells as a subgroup of fibrochondrocytes in condylar cartilage. (A) Schematic representation of sequenced tissue samples from three groups: sham surgery, ADD surgery, and ADD surgery with MagPie implantation. The blue areas indicate the regions of isolated cartilage tissue. (B) UMAP plot of 34 381 cells from cartilage tissue visualizes the general structure colored by 4 major cell types. (C) UMAP plots showing the expression and distribution of marker genes in cells from cartilage tissue. (D) UMAP plot of chondrocytes visualizes the general structure colored by 6 putative cell clusters. (E) Marker gene expression landscape in condylar chondrocyte clusters. (F) Heatmap of cluster‐specific genes among condylar chondrocyte clusters. (G) Pseudotime trajectory of chondrocytes according to the distribution of cell clusters. The color from dark to light indicating the pseudo‐time order. The upward and downward arrows represent the two distinct cell fates. (H) The percentage of different chondrocyte clusters from three groups: sham surgery, ADD surgery, and ADD surgery with MagPie (MP) implantation. (I) UMAP plot showing the expression and distribution of genes encoding voltage‐gated ion channels. The area outlined by the dashed line represents a Prg4^+^ cell population.

To elucidate the cellular and molecular mechanism underlying MagPie‐mediated cartilage repair, we performed scRNA‐seq on cells isolated from rat condylar cartilage. A total of 34381 high‐quality cells were obtained and classified into four major cell clusters, including chondrocytes, macrophages, smooth muscle cells, and endothelial cells (Figure 5A–C, Figure S4). Chondrocytes were subdivided into six clusters: superficial cells‐1 (SFC‐1, expressing *Angptl1*, *Vcan*, and *Dcn*), superficial cells‐2 (SFC‐2, expressing *Prg4*, *Clu*, and *Igfbp5*), proliferative cells (ProC, expressing *Top2a*, *Mki67*, and *Ube2c*), prehypertrophic cells (PreHTC, expressing *Aspn*, *Tnc*, and *Trrc15*), hypertrophic cells‐1 (HTC‐1, expressing *Cdkn1c*, *Panx3*, and *Hapln1*), and hypertrophic cells‐2 (HTC‐2, expressing *Col10a1* and *Ibsp*) (Figure 5D–F). Pseudotime trajectory analysis revealed the progression of chondrocyte differentiation. SFC‐1 and SFC‐2 were distributed at the beginning of the trajectory. PreHTC and HTC‐1 existed along the trajectory, whereas ProC and HTC‐2 were mainly distributed at the end, entering two different cell fates (Figure 5G). Gene Ontology (GO) analysis revealed that the clusters of SFC‐1 and SFC‐2, as well as HTC‐1 and HTC‐2, were involved in different biological processes (Figure S5). SFC‐1 was enriched with genes related to mechanical stimulus and cytoskeleton organization, while SFC‐2 was enriched for GO terms featured in extracellular matrix organization, collagen fibril organization, and cartilage development.

*Prg4*, which encodes lubricin, is a recognized marker of fibrocartilage progenitor cells and plays a role in maintaining cartilage homeostasis [34, 35, 36]. Label‐retaining cells (LRC) revealed that a subset of Prg4^+^ cells retained 5‐ethynyl‐2′‐deoxyuridine (EdU) label (Prg4^+^/EdU^+^) in the superficial zone of cartilage (Figure S6A). Approximately 30.67% ± 4.74% of Prg4^+^ cells were quiescent LRCs, and 44.59% ± 2.15% of LRCs were Prg4^+^ (Figure S6B), indicating some Prg4^+^ cells were quiescent, with slow‐cycling, label‐retaining progenitor identity. Immunofluorescence staining showed that Prg4^+^ cells co‐express the stem/progenitor cell markers CD105, CD166, and Thy1, but not the T‐cell marker CD3 (Figure S6C–E, Figure 7C), further supporting their progenitor‐like properties. Single‐cell transcriptomics analysis showed that *Prg4* expression was specifically enriched in the SFC‐2 cluster (Figure 5E). Based on the expression pattern, we defined SFC‐2 as a Prg4^+^ cell population and inferred that these cells possess reparative functions. Notably, the proportion of Prg4^+^ cells decreased following ADD‐induced degeneration but was restored upon MagPie implantation, suggesting that MagPie treatment expanded Prg4^+^ cells in association with cartilage repair (Figure 5H).

Given that voltage‐gated ion channels mediate electrical signal transduction, we next examined their mRNA expression and found that genes encoding these channels, such as *Scn1a, Cacna1g, Kcnc4*, and *Kcnd1*, were highly expressed in the Prg4^+^ cell population (Figure 5I). These findings imply that Prg4^+^ chondroprogenitor‐like cells are activated after MagPie implantation and that this population exhibits molecular features (e.g., expression of voltage‐gated ion channels) consistent with electrical sensitivity. To test whether Prg4 expression reflects electrical‐sensitive, RPLLA group and Mg^2^^+^‐only treatment group were added in vivo and in vitro for comparison with MagPie. For the in vivo experiment, neither RPLLA nor Mg^2^^+^ treatment significantly altered the number of Prg4^+^ cells, while the MagPie group had a marked increase in Prg4^+^ cells (Figure S6C–E). In vitro, we cultured condylar chondrocytes under applied cyclic pressure. Consistently, only MagPie treatment significantly upregulated Prg4 expression at both mRNA and protein levels compared with other conditions (Figure S7). Together, these findings suggest Prg4^+^ chondroprogenitor‐like cells as a key cell population that contributes to cartilage repair in response to the combined cues provided by MagPie, including its piezoelectric component.

### 2.6 Dbp Is Associated with SFC‐to‐Prg4^+^ Progenitor Differentiation

**FIGURE 6:**
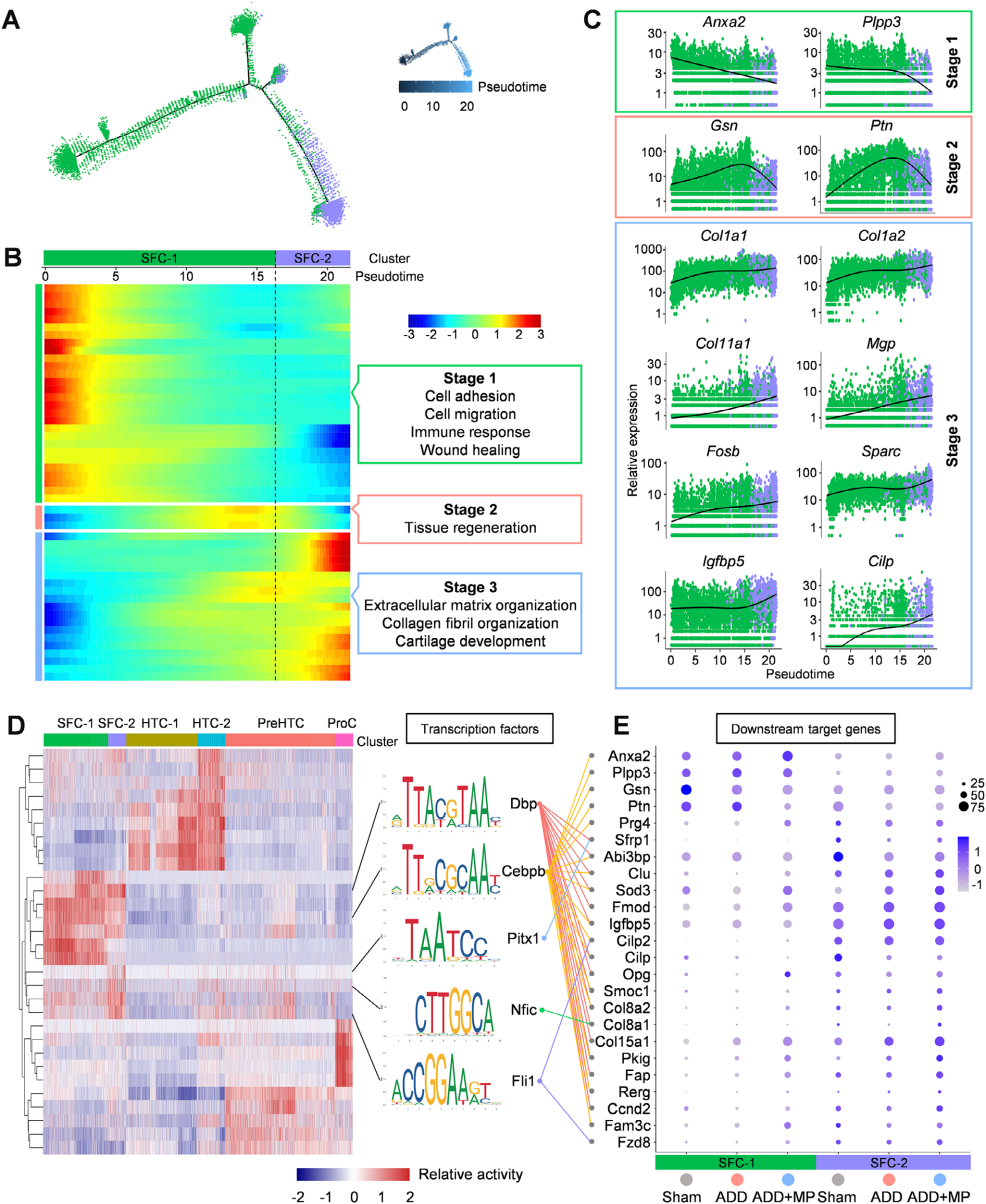
Dbp is associated with SFC‐to‐Prg4^+^ progenitor differentiation. (A) Trajectory plot of fibrochondrocyte clusters, color‐coded by cluster (left) and pseudotime (right). Each dot represents a single cell. (B) Heatmap showing dynamic gene expression changes ordered by pseudotime, illustrating multiple progressive waves of gene expression from SFC‐1 to SFC‐2. Major enriched GO terms for each stage are labeled in right boxes. (C) Curve plots showing expression level changes of selected genes related to each stage along pseudotime. Point colors correspond to cluster colors in (A). (D) Heatmap depicting the average regulon activity in each cluster of chondrocytes. Representative transcription factors are highlighted along with corresponding DNA‐binding motifs. (E) Dot plots showing expression patterns of downstream target genes related to transcription factors in (D). Data are presented from three groups: sham surgery, ADD surgery, and ADD surgery with MagPie (MP) implantation.

Prg4^+^ cells residing at the embryonic joint surface serve as progenitors for deeper layers of mature articular cartilage and play essential roles in joint lubrication and cartilage protection [36, 37, 38]. To trace the cellular origin and differentiation dynamics of Prg4^+^ cells during TMJOA cartilage repair, we performed Monocle pseudotime analysis to delineate the dynamic developmental progression of SFCs. The analysis indicated that Prg4^+^ cells arise from the SFC‐1 population and subsequently give rise to two distinct differentiation lineages (Figure 5G, Figure 6A). To further characterize this transition, we generated a heatmap to analyze dynamic expression changes of differentially expressed genes (DEGs) along the differentiation trajectory from SFC‐1 to SFC‐2 (Prg4^+^ cells) state (Figure 6B). We also classified DEGs into three distinct stages across pseudotime. The initial stage (Stage 1) was marked by significant enrichment of genes associated with cell adhesion, migration, and immune responses. The intermediate stage (Stage 2) showed upregulation of genes related to tissue regeneration, while the late stage (Stage 3) was characterized by increased expression of chondrogenesis‐related genes, indicating a phenotypic shift of these SFCs toward cartilage reparative programs (Figure 6B,C).

To dissect the gene regulatory network regulating SFC differentiation, we used single‐cell regulatory network inference and clustering (SCENIC) to map transcription factor (TF)‐centered gene co‐expression networks [39]. Distinct regulon activity patterns were observed between SFC‐1 and SFC‐2 (Prg4^+^ cells), revealing a complex regulatory network underlying the Prg4^+^ cell state (Figure 6D, Figures S8 and S9). As the pseudotime progressed, the fibrosis‐driving TF Cebpb was highly expressed in SFC‐1, where it positively regulated many downstream target genes in Stage 1 and Stage 2, indicating the inflammatory response status following tissue injury (Figure 6E). In contrast, gene expression of these representative TFs showed Dbp, Pitx1, Nfic, and Fli1 decreased in SFC‐1 but increased in Prg4^+^ cell. Notably, Dbp emerged as a potential key regulator, showing increased regulon activity in Prg4^+^ cells and positively correlating with the expression of chondrogenesis‐associated genes (Figure 6E). These results define a dynamic transcriptional and regulatory framework underlying SFC differentiation and suggest that specific TFs, particularly Dbp, may play a role in facilitating SFCs differentiate into the Prg4^+^ cell state.

### 2.7 MagPie Promotes Prg4^+^ Cells‐mediated Cartilage Repair via the ECM‐FAK‐PI3K‐Akt Axis

**FIGURE 7:**
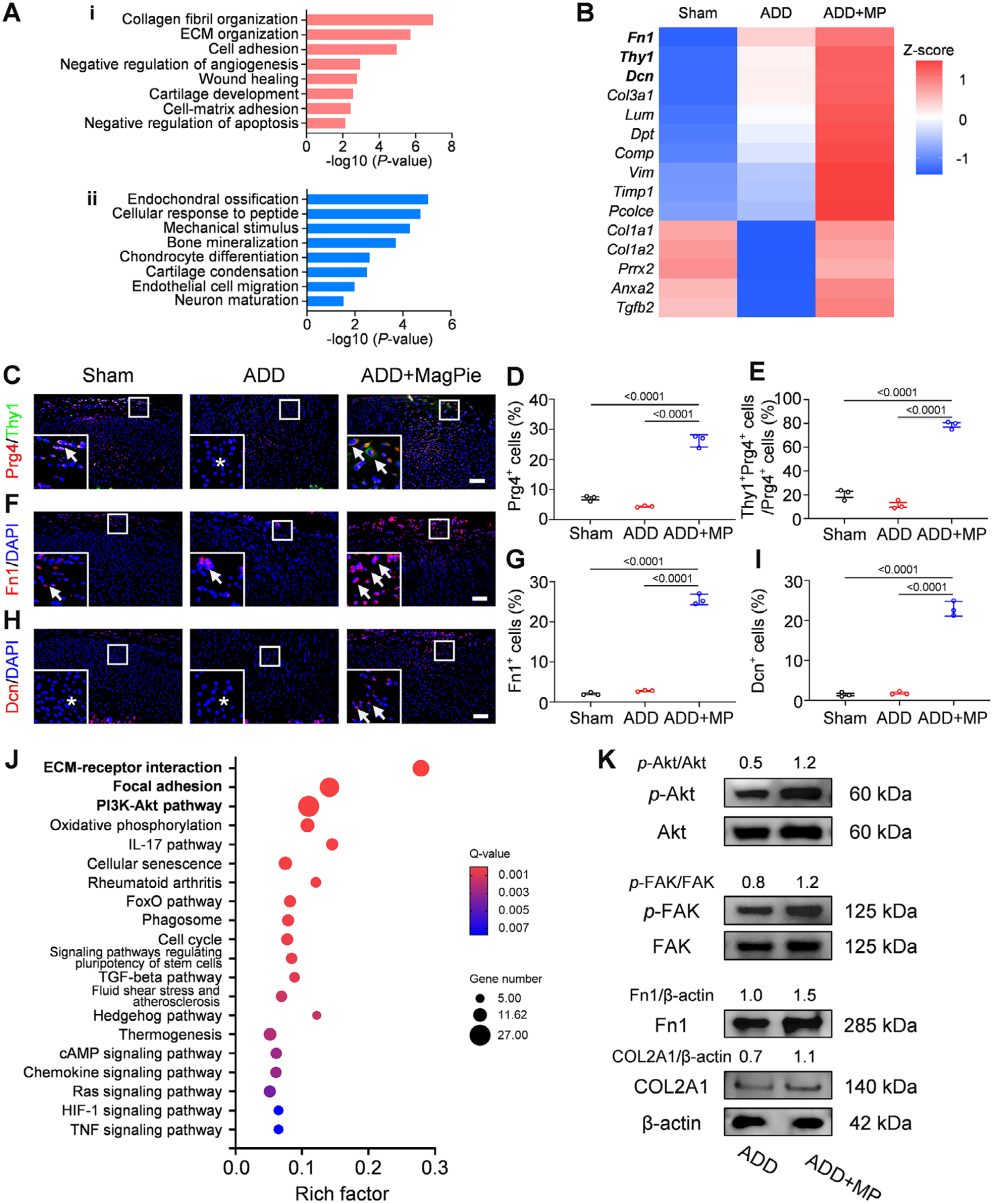
Prg4^+^ cells maintain cartilage homeostasis through the ECM‐mediated FAK‐PI3K‐Akt pathway. (A) GO analysis of Prg4^+^ cells revealed distinct biological process enrichment patterns: up‐regulated (i) and down‐regulated DEGs (ii) when comparing ADD+MagPie (MP) group versus ADD group. (B) Heatmap of Prg4^+^ cell DEGs from different groups. (C) Representative images of Prg4 positive cells (red) and Thy1 positive cells (green) in TMJ sagittal sections. The white arrows indicate positive cells, and the asterisks indicate negative signals in cartilage. Scale bar: 100 µm. (D,E) Quantitative analysis of the percentage of Prg4 positive cells and Prg4 & Thy1 double positive cells. Data were presented as means and SEM. *n* = 3 per group. (F,G) Representative images and quantitative analysis of Fn1 positive cells. The white arrows indicate positive cells in cartilage. Scale bar: 100 µm. Data were presented as means and SEM. *n* = 3 per group. (H,I) Representative images and quantitative analysis of Dcn positive cells. The white arrows indicate positive cells, and the asterisks indicate negative signals in cartilage. Scale bar: 100 µm. Data were presented as means and SEM. *n* = 3 per group. (J) KEGG analysis showed distinct pathway enrichment in Prg4^+^ cell DEGs between the ADD group and the ADD+MagPie group. (K) Representative Western blotting bands showing the expression of *p*‐Akt, Akt, *p*‐FAK, FAK, Fn1, COL2A1, and β‐actin in cartilage in the ADD group and the ADD+MagPie group.

To further explore the potential roles of the Prg4^+^ cell population after MagPie treatment, we screened DEGs of SFC‐2 between the ADD group and the ADD+MagPie group. Compared with the ADD group, DEGs in the MagPie group were enriched in processes related to collagen fibril organization, extracellular matrix assembly, and cartilage development, while pathways associated with endochondral ossification, angiogenesis, and neuron maturation were suppressed, consistent with our in vivo observations (Figure 7A). In addition, the vital ECM proteoglycan‐related genes *Fn1* and *Dcn*, the mesenchymal stem cell marker *Thy1*, along with the fibrocartilage markers *Col1a1* and *Col1a2* were highly enriched in the cluster SFC‐2 in the MagPie group (Figure 7B). Immunofluorescent staining confirmed an elevated proportion of Prg4^+^ cells upon MagPie implantation. Moreover, a higher fraction of Prg4^+^ cells co‐expressed Thy1 in the MagPie group compared with both sham and ADD groups (Figure 7C–E). Consistently, Fn1 and Dcn expression levels were significantly increased in the MagPie group, and they were mainly distributed in the superficial zone of the cartilage (Figure 7F–I). These findings indicate that the Prg4^+^ cells function as chondroprogenitor‐like cells in cartilage repair and exhibit enhanced ECM synthetic capacity following MagPie treatment.

Kyoto Encyclopedia of Genes and Genomes (KEGG) pathway analysis was conducted to reveal the possible underlying chondrogenic signaling pathways in which the DEGs were enriched. ECM‐receptor interaction, focal adhesion, and PI3K‐Akt pathway, were notably affected by MagPie stimulation (Figure 7J). PI3K‐Akt is an important intracellular pathway that can synergize with its upstream ECM‐receptor interaction pathway and focal adhesion pathway to regulate cell proliferation, migration, and protein synthesis. The importance of PI3K‐Akt pathway on cartilage regeneration has been proved by some studies [40, 41, 42, 43]. As shown in Western blot, the expression levels of phosphorylated FAK (*p*‐FAK) and phosphorylated Akt (*p*‐Akt), which are key molecules in the focal adhesion pathway and the PI3K‐Akt pathway, respectively, were upregulated in the MagPie group. Similarly, the expression of the ECM proteoglycan Fn1 and COL2A1 is also upregulated (Figure 7K). In summary, these results suggest that MagPie promotes Prg4^+^ cell anabolism, potentially involving the ECM‐mediated FAK‐PI3K‐Akt pathway (Figure S10).

### 2.8 MagPie Modulates Prg4^+^ Cell‐macrophage Crosstalk to Maintain Cartilage Homeostasis

**FIGURE 8:**
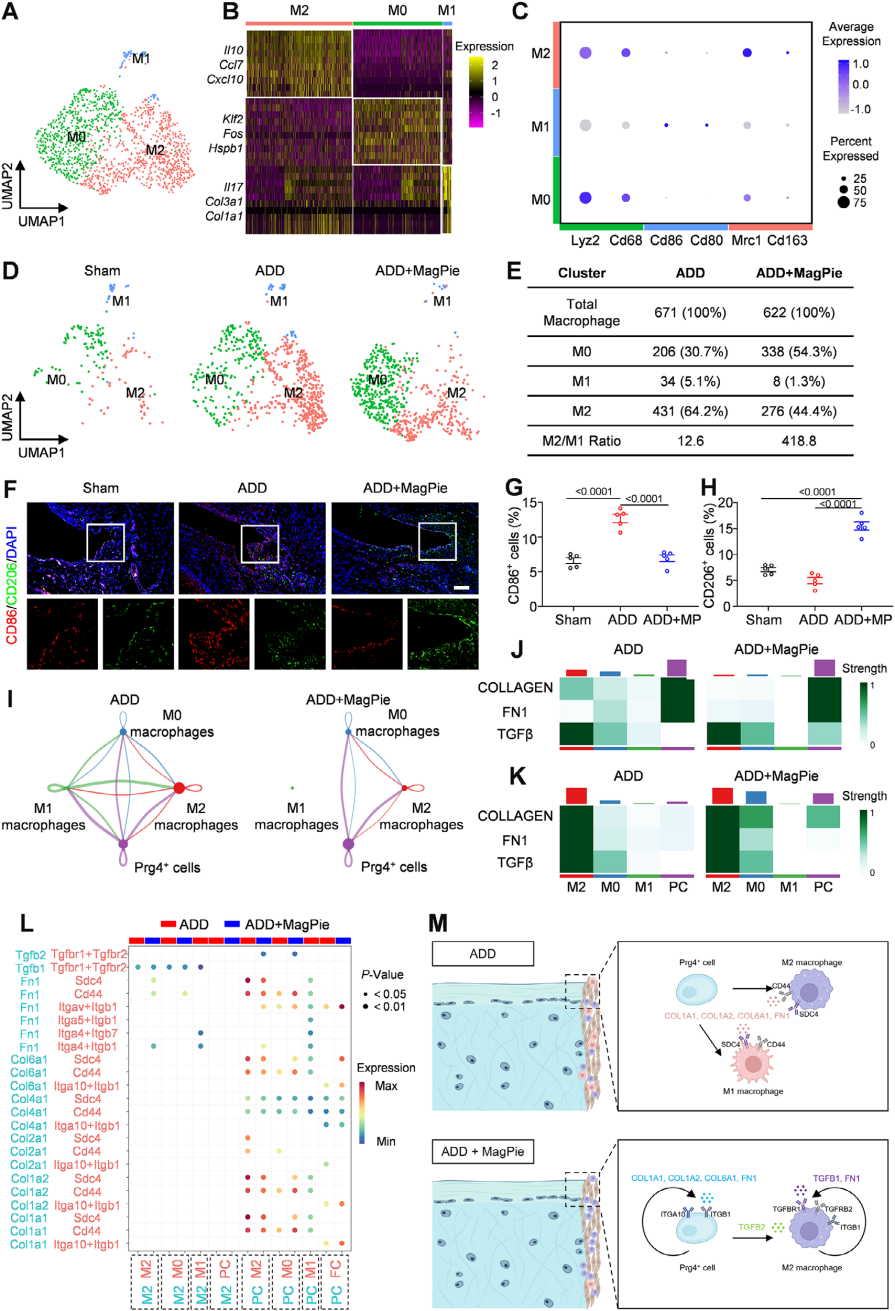
MagPie modulates macrophage‐Prg4^+^ cell crosstalk to maintain cartilage homeostasis. (A) UMAP plot of macrophages visualizes the general structure, colored by three putative cell clusters. (B) Heatmap showing differentially expressed genes among M0, M1, and M2 macrophage phenotypes. (C) Dot plot showing the expression of representative M0, M1, and M2 marker genes in each macrophage cluster. (D) UMAP visualization of macrophage distribution across experimental groups: sham surgery (left), ADD surgery (middle), and ADD surgery with MagPie implantation (right). (E) Number and proportion of M0, M1, and M2 macrophages in the ADD and ADD+MagPie groups. (F) Representative images of CD86‐positive cells (red, represented M1 macrophages) and CD206‐positive cells (green, represented M2 macrophages) in TMJ sagittal sections from three groups: sham, ADD, and ADD+MagPie. The white arrows indicate positive cells in condylar cartilage. Scale bar: 100 µm. (G) Quantitative analysis of the percentage of CD86‐positive macrophages. Data were presented as means and SEM. *n* = 5 per group. (H) Quantitative analysis of the percentage of CD206‐positive macrophages. Data were presented as means and SEM. *n* = 5 per group. (I) Interaction number of incoming and outgoing events between Prg4^+^ cells and all macrophage phenotypes in the ADD and ADD+MagPie groups. (J) Heatmap showing outgoing signaling patterns associated with chondrogenesis‐related pathways in Prg4^+^ cells and all macrophage phenotypes across ADD and ADD+MagPie groups. (K) Heatmap showing ingoing signaling patterns associated with chondrogenesis‐related pathways in Prg4^+^ cells (PC) and all macrophage phenotypes across ADD and ADD+MagPie groups. (L) Dot plot generated by Cell Chat showing potential ligand‐receptor pairs associated with COLLAGEN, FN1, and TGFβ signaling between Prg4^+^ cells and all macrophage phenotypes in the ADD and ADD+MagPie groups. Dots colored by the mean expression of ligand‐receptor pair between two clusters, and dots size proportional to *p*‐Value. (M) Schematic illustration depicting potential crosstalk between Prg4^+^ cells and macrophages.

Synovial macrophages surrounding the condylar cartilage play a critical role in regulating the joint microenvironment. To evaluate the immunomodulatory effect of MagPie in the arthritic microenvironment, macrophages were classified into three subpopulations: resting (M0), inflammatory (M1), and anti‐inflammatory (M2) phenotypes (Figure 8A–C). The results revealed a significant increase in all macrophage subpopulations following ADD compared to the sham group (Figure 8D). After MagPie implantation, both the number of M0 macrophage and the ratio of M2/M1 macrophage were elevated (Figure 8E), indicating a shift toward an anti‐inflammatory phenotype. Immunofluorescence staining of whole TMJs found that macrophages were primarily localized at the synovium‐cartilage junction and were markedly increased in the ADD group (Figure 8F), indicating that the macrophages identified by scRNA‐seq were predominantly synovium‐derived. The results also revealed a shift in macrophage polarization from M1 to M2 in the MagPie group, indicating a reparative state induced by MagPie stimulation (Figure 8F–H).

Based on CellChat analysis to infer intercellular communications of the co‐localized cell subpopulations [44], we investigated potential interactions between Prg4^+^ cells and macrophages. The results identified Prg4^+^ cells emerged as the dominant communication hub, which secreted and received signals via 217 and 120 ligand‐receptor pairs, respectively. In the ADD group, Prg4^+^ cells interacted with all three macrophage states; whereas in the MagPie group, interactions were predominantly restricted to M0 and M2 macrophages (Figure 8I). Compared with the ADD group, Prg4^+^ cells in the MagPie group mediated more efferent events in chondrogenesis‐related pathways (Figure 8J). M2 macrophages participated in the most afferent events in these pathways, possibly representing a receiving center in regulating the arthritic microenvironment (Figure 8K). Prg4^+^ cells in the ADD group expressed relatively high levels of COLLAGEN signaling ligands, and the corresponding receptors Sdc4 and Cd44 were apparently increased in M1‐ and M2‐ macrophages (Figure 8L,M). Prg4^+^ cells in the MagPie group exhibited higher expression of *Tgfb2* and *Fn1*, raising the possibility that they recruit and interact with M2 macrophages via *Tgfb2*/*Tgfbr1*, *Tgfb2*/*Tgfbr2*, and *Fn1*/*Itgb1* during cartilage repair (Figure 8L,M).

### 2.9 Loss of Prg4 Compromises MagPie‐mediated Therapeutic Effects in TMJOA

**FIGURE 9:**
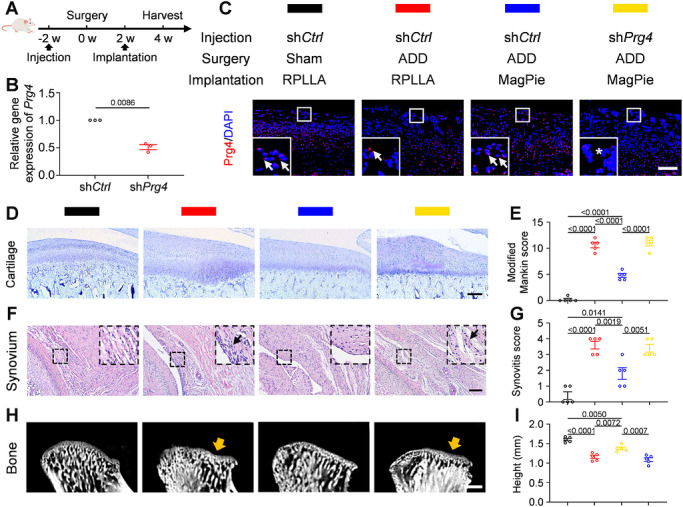
Loss of Prg4 compromises MagPie‐mediated therapeutic efficacy in TMJOA. (A) Schematic diagram of the animal experiment operation protocol. (B) The efficiency of sh*Prg4* examined via real‐time qPCR. Data were presented as means and SEM. *n* = 3 per group. (C) The efficiency of sh*Prg4* examined via immunofluorescence staining. The white arrows indicate positive cells, and the asterisks indicate negative signals in cartilage. Scale bar: 100 µm. (D) Representative images of toluidine blue (TB) staining. Scale bar: 200 µm. (E) Quantitative analysis of the modified Mankin score of condyles. Data were presented as means and SEM. *n* = 5 per group. (F) Representative images of HE staining. Scale bar: 100 µm. (G) Quantitative analysis of synovitis score. Data were presented as means and SEM. *n* = 5 per group. (H) Micro‐CT images of TMJ condyles. The yellow arrows indicate condylar surface flattening and local sclerosis. Scale bar: 1 mm. (I) Quantitative analysis of the height of TMJ condylar. Data were presented as means and SEM. *n* = 5 per group.

To further clarify the role of Prg4 in condylar cartilage repair and TMJOA treatment, 8‐week‐old male SD rats received intra‐articular injection of adeno‐associated virus (AAV) carrying Prg4‐specific shRNA (sh*Prg4*) or the vector control (sh*Ctrl*) (Figure 9A). Efficient knockdown of sh*Prg4* in cartilage was verified by real‐time qPCR and immunofluorescence staining (Figure 9B,C). In the sham group, rats injected with sh*Ctrl* showed intact cartilage and normal condylar morphology. After ADD surgery, cartilage degradation was obvious, evidenced by markedly higher modified Mankin scores. Although MagPie implantation significantly attenuated TMJOA progression in rats following ADD surgery, sh*Prg4* administration counteracted its therapeutic benefits, resulting in pronounced cartilage degradation and synovial inflammation (Figure 9D–G). Consistently, Micro‐CT showed that MagPie alleviated subchondral bone sclerosis and osteophyte formation at 4 weeks after ADD surgery, but this effect was eroded by *Prg4* knockdown (Figure 9H). Similarly, condylar head height was significantly increased after MagPie implantation, but knockdown of *Prg4* weaken the increase of condylar head height significantly (Figure 9I). Thus, Prg4 downregulation diminishes the therapeutic efficacy of MagPie and accelerates TMJOA development, supporting the importance of Prg4^+^ cells in MagPie‐mediated cartilage repair.

## 3 Discussion

Herein, we demonstrated that the biodegradable MagPie exerted synergistic therapeutic effects under mastication by integrating piezoelectric stimulation, Mg^2^^+^ release, and pH modulation, thereby promoting pro‐regenerative and anti‐inflammatory responses in condylar chondrocytes. Under excessive joint loading, MagPie activated Prg4^+^ cells to promote cartilage repair. Single‐cell transcriptomics revealed that Prg4^+^ cells exhibited progenitor‐like properties and electro‐responsiveness, playing a key role in maintaining joint homeostasis. Mechanistically, MagPie enhanced the anabolic activity of Prg4^+^ cells via the ECM‐mediated FAK‐PI3K‐Akt pathway. Furthermore, Prg4^+^ cells modulated the arthritic microenvironment by promoting macrophage polarization toward an anti‐inflammatory phenotype. This study thereby outlines a “trash‐to‐treasure” strategy that leverages harmful joint overloads to generate beneficial electrical cues and Mg^2+^ release at the condylar surface, thus improving the TMJ osteoarthritic microenvironment and facilitating cartilage repair.

Given the electrical sensitivity of cartilage, piezoelectric materials have been developed to generate electrical stimulation for cartilage regeneration. Our previous study found that PLLA films under applied force enhanced cartilage regeneration in a rabbit knee osteochondral defect model [29]; however, PLLA alone failed to mitigate degenerative changes in condylar cartilage. To address the inflammatory microenvironment of the TMJ, we incorporated MgO into PLLA film to neutralize acidic degradation by‐products, resulting in a hybrid design—MagPie—that effectively promoted chondrocyte anabolism and facilitated condylar cartilage repair in ADD‐induced TMJOA rats. This approach may offer clinical potential, for example, through arthroscopic implantation of MagPie onto the condylar surface to directly target cartilage degeneration and slow disease progression. Although in vitro experiments included PLLA and Mg^2^^+^ groups, the scRNA‐seq and in vivo comparisons involved conditions differing in piezoelectric behavior, Mg^2^^+^ release, and pH modulation. Therefore, the data support the overall efficacy of MagPie as a multifunctional system driven by the synergy of these factors. Future studies employing control materials matched in all properties except piezoelectricity are required to delineate the specific contribution of each parameter.

Anatomically, TMJ condylar cartilage is a fibrocartilaginous tissue with a distinct zonal architecture: the superficial zone (SFZ) is enriched in type I collagen and harbors flattened stem/progenitor cells, while the deeper zones are predominantly populated by differentiated chondrocytes that produce type II collagen, the primary marker of the mature cartilage matrix [45, 46]. Prg4^+^ cells serve as the dominant chondroprogenitors within the SFZ, capable of differentiating into chondrocytes to maintain cartilage homeostasis and support intrinsic cartilage regeneration [34, 35, 36]. Proteoglycan 4 (PRG4), secreted by Prg4^+^ cells, plays a critical role in maintaining articular cartilage integrity [47]. Deficiency of PRG4 in both humans and mice resulted in early‐onset OA, characterized by loss of superficial zone cells and synovial hyperplasia [37]. During OA progression, PRG4 gradually decreased after a transient elevation, which is related to stem/progenitor cell pool depletion and subsequent cartilage degeneration [48]. Therapeutic strategies targeting PRG4 in OA have shown promising results, including recombinant PRG4 protein injections, gene therapies, and small molecules that enhance endogenous Prg4 expression or mimic its function [49, 50]. Our results recapitulated this pattern in the ADD‐induced TMJOA model. Under mechanical overloading caused by ADD, Prg4^+^ cells decreased and closely correlated with cartilage degradation. Notably, a recent study indicated that Procr^+^ chondroprogenitors, a subset of Prg4^+^ cells, could sense mechanical stimuli, enabling targeted cartilage regeneration through external biophysical cues [22]. In this study, scRNA‐seq further identified Prg4^+^ cells as a cell population with molecular features consistent with electrical sensitivity. MagPie implantation significantly expanded the Prg4^+^ cell population and promoted type II collagen expression in cartilage, suggesting that electrical stimulation may drive Prg4^+^ cells toward a mature chondrocyte phenotype. Collectively, these findings highlight the activation of Prg4^+^ cells via electrical stimulation as a promising and precise therapeutic strategy for TMJOA.

In conclusion, this study proposes a ‘trash‐to‐treasure’ strategy that harnesses pathological joint overloading to activate endogenous Prg4^+^ cells for OA treatment. Despite these promising findings, several limitations warrant consideration: (i) direct in vivo quantification of output voltage is needed to establish the relationship between voltage magnitude and therapeutic efficacy; (ii) isolation and characterization of Prg4^+^ cells are required to confirm their multilineage potential and to support the development of Prg4^+^‐based regenerative strategies; (iii) precise mechanisms by which MagPie regulates Prg4^+^ cell fate requires further elucidation in vitro and in vivo; (iv) the experiments was conducted in an early‐stage, short‐term male rat model of ADD‐induced TMJOA, which may limit the generalizability of the findings. Future studies employing Prg4 transgenic models and exogenous Prg4^+^ cell transplantation will be essential to determine whether the MagPie‐responsive Prg4^+^ population represents the principal effector cell type and to further define the underlying regulatory mechanisms. In addition, long‐term evaluations across both sexes, different age groups, and more advanced disease stages are needed to fully assess the translational potential of this strategy.

## 4 Materials and Methods

### 4.1 Fabrication of the Biodegradable Piezoelectric Films

PLLA was dissolved in dichloromethane, while MgO was dispersed in dimethylformamide via ultrasonication. Prior to electrospinning, the PLLA solution was thoroughly mixed with 5% MgO dispersion. The piezoelectric PLLA‐MgO films (termed as MagPie) and PLLA films were fabricated at a drum speed of ∼4000 rpm, whereas the nonpiezoelectric RPLLA films were fabricated at ∼300 rpm, with an applied voltage of 13.3 kV and a feeding rate of 0.12 mm/min. The obtained films were annealed under 110°C for 10 h and cooled down to room temperature, followed by a second annealing step at 140°C for 10 h. And the films were sterilized by ^60^Co before being applied to cells and animals.

### 4.2 Structural and Mechanical Characterization

The morphology and elemental composition of the nanofibers were characterized by scanning electron microscopy (SEM) and energy‐dispersive X‐ray spectroscopy (EDS) (JSM‐7900F, JEOL Ltd, Japan). Transmission electron microscopy (TEM) (JEM‐F200, JEOL Ltd, Japan) was used to observe the nanofiber structure. X‐ray diffraction (XRD) patterns were recorded using X‐ray diffraction (D8 Advance, Bruker, Germany). The elastic modulus was measured using a universal testing system (INSTRON, USA).

### 4.3 Finite Element Analysis

To simulate the stress distribution of piezoelectric materials implanted in the temporomandibular joint and subsequently calculate the induced voltage, a quasi‐static mechanical compression model was established in Abaqus/Standard for finite element analysis. The mean pressure of the anterior surface of condyles generated by disc displacement (931.72 ± 56.50 kPa) was referred to a previous study [30]. The geometries of the temporomandibular joint and the piezoelectric material were reconstructed from CT data obtained 4 weeks post‐implantation, followed by finite element meshing. A constant‐velocity displacement was applied to the temporal bone to ensure close contact between the piezoelectric material and the condylar. In the post‐processing stage, von Mises stress was employed to evaluate the overall loading condition and stress distribution of the piezoelectric material under varying compression levels. The output voltage was further calculated according to the formula based on the published data (V = 0.0097 * P – 0.1738).

### 4.4 Cell Isolation and Expansion

Primary chondrocytes were isolated by dissecting condylar cartilage from neonatal rats. Specifically, the condylar cartilage tissue was isolated, thoroughly cleaned, and cut into small explants, of approximately 1 mm^3^ in size. The cartilage explants were spaced evenly and cultured in Dulbecco's modified Eagle medium (DMEM; Gibco, USA) supplemented with 20% fetal bovine serum (FBS; Gibco) and 1% penicillin‐streptomycin (Gibco) at 37°C in a humidified 5% CO_2_ atmosphere. The culture medium was partially changed every 3 days. About 7 days later, chondrocytes migrated out of the explants and attached to the surface of the dish, forming a monolayer or colony of cells. Then, chondrocytes were trypsinized and replated at a lower density to expand the cell population.

### 4.5 In Vitro Pressurized Cell Culture

All pressurized cell culture experiments were conducted using a custom‐built pressure chamber bioreactor (NK‐700, Naturethink Life Technology Ltd., China). Chondrocytes were seeded into the pressure chamber at 37°C with 5% CO_2_ atmosphere and then covered with or without biodegradable films. Cyclic hydrostatic pressure at a frequency of 1 Hz was applied in the chamber by a numerically controlled apparatus. The controls were seeded into the same chamber and cultured without pressure.

### 4.6 Animal Model

Eight‐week‐old male Sprague‐Dawley rats (260–280 g, Vital River Laboratory, China) were randomly assigned into five groups: sham, ADD, ADD with RPLLA film, ADD with Mg^2+^, and ADD with MagPie. All animals were kept in a specific pathogen free environment with a 12‐h light/dark cycle at room temperature and given food and water ad libitum. For euthanasia, CO_2_ (1.5–2.0 L/min) was pumped into sealed cages to sacrifice the rats. Cervical dislocation was used to verify the death of experimental rats. All animal care and procedures were performed in compliance with animal welfare ethical regulations and approved by the Peking University Animal Ethics Committee (PUIRB‐LA2023269). The surgical operations were carried out according to our previously established procedures [30]. Rats were anesthetized by intraperitoneally injecting 1% pentobarbital sodium. A sterile silk suture (5–0) was threaded through the posterior band of the disc vertically and anchored to the bend point of the zygomatic arch, thereby repositioning the disc from its original position on the upper surface to the anterior surface of the condyle to induce anterior disc displacement. In the sham group, the disc was separated from the condyle without displacement. In the Mg^2^^+^ treatment group, the rats at 2 weeks after ADD surgery received intra‐articular injections of MgCl_2_ (8.8 mg/L, once daily, 2 weeks) to mimic Mg^2^^+^ release from MagPie.

### 4.7 In Vivo Biodegradable Film Implantation

Two weeks after surgery, the rats received the implantation of biodegradable film under anesthesia. MagPie or RPLLA film of the same size (2 mm × 3 mm) as the anterior surface of condyle was implanted in the lower compartment of articular cavity. This location is the load‐bearing surface of the temporomandibular joint and was chosen so that the film could experience the pressure on the anterior surface of condyle. The sham group and the ADD group operated similarly but without film implantation. After operation, the wound was rinsed, the muscles and skin were sutured in layers. Fentanyl patch was applied once to provide analgesic relief. The daily food intake was recorded to observe the usage of TMJs. The rats were euthanized at 2 weeks after implantation, and the TMJ samples were harvested for subsequent experiments.

### 4.8 Label‐retaining Cells (LRCs) Experiment

LRC is a well‐established approach for identifying quiescent stem cells in different tissues [51, 52]. Pregnant wild‐type mice were injected with EdU daily for 5 days at a dosage of 25 µg/g before delivery and analyzed for another 8 weeks. Edu was detected using a Click‐iT Plus EdU Cell Proliferation Kit (Invitrogen, C10337). Immunofluorescence staining was performed to visualize the co‐localization of Prg4^+^ cells and EdU^+^ cells.

### 4.9 Single‐cell RNA Sequencing

According to the manufacturer's protocol, single‐cell suspensions were loaded to the Chromium Single‐Cell platform for single‐cell capture, cDNA amplification, and library construction using the Chromium Single Cell 3’ Reagent kit (version 3; 10× Genomics, USA). The libraries were sequenced on an Illumina NovaSeq 6000 sequencing system with a sequencing depth of at least 20000 reads per cell with a paired‐end 150 bp reading strategy (performed by LC‐Bio Technology Co., Ltd., China).

### 4.10 Preparation of Single‐cell RNA Suspensions

A total of 18 rats from the sham, ADD, and ADD with piezoelectric film groups (*n* = 6) were used in this experiment. After rats were euthanized, cartilage samples were carefully dissected from condylar heads under a stereomicroscope for obtaining intact tissue. Given the anatomical continuity of marginal cartilage with the overlying synovium, a minimal amount of synovial tissue might be inevitably included. The tissue was cut into small pieces, repeatedly washed with PBS, and digested at 37°C for 2 h in 10 mL of DMEM containing 0.2% NB4 collagenase and 0.3 mg/mL DNase I (Thermo Scientific). The mixture was filtered through a 40‐µm cell strainer and centrifuged at 300 g for 5 min at 4°C. Then the cell pellets were resuspended in 50 µL of PBS (0.04% BSA). The overall cell viability was confirmed by trypan blue staining, which needed to be above 85% for further processing. Single cell suspensions were counted using a hemocytometer, and about 15 000 cells per group were used for experiments.

### 4.11 Data Processing for scRNA‐seq

Raw sequencing data were aligned to the Rattus norvegicus Ensembl reference genome (version 110), and estimated cells and associated unique molecular identifiers (UMIs) were counted using the CellRanger Single‐Cell Software Suite (version 7.2.0, 10× Genomics). Downstream analyses were performed with the R software package Seurat version 4.1.0. First, cells with a gene number of < 500, or a mitochondrial gene ratio of >25% were regarded as abnormal and filtered out. Then, the FindVariableFeatures function was employed to identify 2000 highly variable genes, and the LogNormalize function was used to normalized data. Subsequently, principal component analysis was performed on the regressed highly variable genes, selecting the top 20 PCs for clustering by the FindClusters function. For each cluster, differentially expressed genes were identified using the FindMarkers function. And data visualization was realized by TSNE and UMAP. The KEGG and GO enrichment were carried out by the OmicStudio tool (https://www.omicstudio.cn/cell), with a *p*‐value < 0.05 as significant enrichment. The results were visualized in R.

### 4.12 Statistical Analysis

Data were expressed as mean ± standard error of mean (SEM). Sample size was presented in the figure legends. Each result shown in the representative images has been verified by at least three independent experiments with biological replicates. One‐way ANOVA with post hoc Bonferroni's multiple comparison test or unpaired *t* test was employed to analyze these data sets (GraphPad Prism software 10.0, USA). Differences were considered statistically significant at *p* values < 0.05.

## Author Contributions

S.Y.F.: Conceptualization, Methodology, Investigation, Visualization, Funding acquisition, and Writing – original draft. J.R.C.: Conceptualization, Methodology, Investigation, Visualization, and Writing – original draft. Y.Q.: Methodology, Investigation, and Visualization. J.L.: Investigation, Visualization, and Funding acquisition. C.C.G., H.Y.F., and L.P.W.: Methodology and Investigation. T.D.N.: Supervision. Y.L., K.Y.F., and X.L.D.: Conceptualization, Funding acquisition, Project administration, Supervision, and Writing – review & editing. The authors acknowledge the use of ChatGPT 5.3 for language editing to improve the clarity and readability of the manuscript.

## Conflicts of Interest

The authors declare no conflicts of interest.

## Supporting information




**Supporting File**: advs75725‐sup‐0001‐SuppMat.docx.

## Data Availability

The data that support the findings of this study are available in the supplementary material of this article.
