# Supplementary material for: Mastication‐Induced Electrical Stimulation Activates Prg4+ Chondroprogenitors for Osteoarthritis Therapy
Source: Adv Sci (Weinh). 2026 May 19;13(45):e75725. doi: 10.1002/advs.75725 (PMC13336053; doi:10.1002/advs.75725)
Supplement: Supplementary file 1 [file ADVS-13-e75725-s001.docx]

**Supplementary Materials for**

**Mastication-induced electrical stimulation activates Prg4⁺ chondroprogenitors for osteoarthritis therapy**

Shi-Yang Feng^1,3,4,5#^, Jing-Rong Cheng^2,3,4,5#^, Yu Qin^6^, Jie Lei^1,3,4,5^, Chen-Chen Gao^1,3,4,5^, Hong-Ying Fu^2,3,4,5^, Li-Ping Wu^2,3,4,5^, Thanh D. Nguyen^7,8,9^, Xu-Liang Deng^2,3,4,5*^, Kai-Yuan Fu^1,3,4,5*^, Yang Liu^2,3,4,5*^

^1^Center for TMD & Orofacial Pain, Peking University School and Hospital of Stomatology; Beijing, 100081, China.

^2^Department of Dental Materials & Dental Medical Devices Testing Center, Peking University School and Hospital of Stomatology; Beijing, 100081, China.

^3^NMPA Center for Innovation and Research in Regulatory Science & Beijing Key Laboratory of Digital Stomatology, National Engineering Research Center of Oral Biomaterials and Digital Medical Devices; Beijing, 100081, China.

^4^National Center for Stomatology & National Clinical Research Center for Oral Diseases & NHC Research Center of Engineering and Technology for Computerized Dentistry; Beijing, 100081, China.

^5^Beijing Key Laboratory of Biomaterials for Oral Disease, Peking University School and Hospital of Stomatology; Beijing, 100081, China.

^6^Department of Orthopedics, Peking University Third Hospital; Beijing, 100191, China.

^7^Department of Biomedical Engineering, University of Connecticut; Storrs, CT 06269, USA.

^8^Institute of Materials Science, Polymer Program, University of Connecticut; Storrs, CT 06269, USA.

^9^Department of Mechanical Engineering, University of Connecticut; Storrs, CT 06269, USA.

**This file includes:**

Materials and Methods

Fig. S1-S11

Table S1

**Materials and Methods**

**Degradation test and Ion concentration, pH value test**

Put RPLLA, PLLA and MagPie in PBS for 12 weeks, keep monitoring their remaining weight. The pH value was measured through pH-meter in day 0, 3, 6, 9, 12 when materials immersed in PBS. Synovial fluid was collected from TMJOA rats with different materials implanted and control group, then the pH value was measured. And the concentration of Mg^2+^ was measured by ICP-OES (ThermoICPOES7200, ThermoFisher, USA) in day 6, 9, 12.

**Measurement of piezoelectric modulus and output voltage under impact system**

MagPie (50 to 55 µm thick), PLLA films and RPLLA films were cut at a 45° angle with relative to the fiber direction into a 1.1 cm–by–1.1 cm films. The aluminum (Al) foil was cut into 1 cm–by–1 cm square to place on both sides of the film as electrodes. Polyimide tape (3M) was then used to package the sandwich-like structure. The exposed Al foil electrode leads were then reinforced using copper tape. Linear motor (C1XX0, LinMot, Switzerland) was used to apply a constant force at a frequency of 1 Hz on the films. The output voltage was monitored and recorded using an electrometer (Keithley 6514 electrometer, Tektronix Inc).

**Cell viability**

Cell viability was evaluated by the Live/Dead staining and the Cell Counting Kit-8 (CCK-8) assay. In brief, chondrocytes were seeded onto films for 24 hours to fully attach, and ensuring that the films could fully contact the cells. A Live/Dead staining kit (Beyotime, China) was used to assess cell viability on the films. Live cells labeled with Calcein AM for green fluorescence, and dead cells labeled with PI red fluorescence. The cells were then observed under confocal laser scanning microscope (A1R HD25, Nikon, Japan). CCK-8 (Dojindo, Japan) was conducted according to the manufacturer’s instructions. After 24, 48 and 72 hours culturing respectively, the cells were incubating with CCK-8 solution for 2 h, optical density (O.D.) was read at 450 nm using an absorbance microplate reader (ELx808, BioTek, USA).

**Cell migration**

The cells were initially inoculated at a density of 2.5 × 10^6^ with complete culture medium in the 6-well plates. Homogeneous scratching was performed after 24 h of starvation when the cells reached 80%. A 200 μL tip was used to make a scratch on the monolayer of cells. The film was covered for piezoelectric stimulus generation and Mg^2+^ release. Cell distribution images were acquired at 0 and 24 hours after stimulation, and the areas of cell migration were quantified by ImageJ software (Media Cybernetics Inc., USA).

**Real-time quantitative polymerase chain reaction (RT-qPCR)**

Total RNA was isolated from primary chondrocytes using MiniBEST Universal RNA Extraction Kit (9767, TaKaRa, Japan) according to the manufacturer’s instruction, and the concentrations were measured with a NanoDrop 8000 spectrophotometer (Thermo Fisher Scientific, USA). Then the mRNA was converted to single-stranded complementary DNA (cDNA) via PrimeScript RT Master Mix (RR036A, TaKaRa, Japan). RT-qPCR was carried out with the cDNA, primers and SYBR Green reagent (Roche, Switzerland) through 7500 Real-Time PCR System (Thermo Fisher Scientific). The chondrogenic-associated gene *Prg4* was detected with glyceraldehyde-3-phosphate dehydrogenase (*Gapdh*) as the housekeeping gene. The sequences of gene-specific primers were listed in table S1.

**Adeno-associated virus (AAV) infection**

In vivo gene knockdown was achieved by AAV. Construction and production of recombinant AAV Serotype 9 carrying *Prg4* shRNA (sh*Prg4*) or vector control (sh*Ctrl*) were manufactured by GeneChem Company (Shanghai, China). To specifically inhibit the expression of Prg4 in condylar cartilage, sh*Prg4* (or sh*Ctrl*) was injected intra-articularly at a final titer of 2 × 10^12^ transducing units/mL (total volume of 50 μL per rat) on the 2nd week before surgery. The transfection efficiency of AAV was routinely checked by real-time qPCR and immunofluorescence staining.

**Micro-computed tomography (micro-CT) analysis**

The TMJ specimens were fixed in 10% formalin overnight and scanned at 80 kV, 500 μA, and 33.658 μm pixel size with an Inveon micro-CT system (Siemens, Germany). The acquired axial images were reconstructed and imported into the Inveon Research Workplace software for visualization and analysis. The region of interest was defined as the area within 70 consecutive cross-sectional images extending downwards from the apex of condyle, in order to encompass the condylar head region above condylar neck. The BMD and height of the condylar head for each sample were calculated.

**Histological, TUNEL, and immunofluorescence staining**

The TMJ specimens were fixed in 10% formalin, decalcified in 10% EDTA at a pH of 7.4, and subsequently embedded in paraffin. The sagittal sections of TMJs with 5-μm in thickness were prepared and processed for staining. Hematoxylin-eosin (HE; Solarbio, China), safranin O-fast green (SOFG; Solarbio), toluidine blue (TB; Solarbio), and TUNEL (Roche) staining were performed according to standard procedures.

For immunofluorescence staining, tissue sections were blocked and incubated with the primary antibodies overnight at 4°C, including anti-COL2A1 (1:200; Novus, USA), anti-MMP3 (1:100; Proteintech, China), anti-MMP13 (1:100; Proteintech), anti-iNOS (1:100; Abcam, USA), anti-Prg4 (1:100; Novus), anti-CD105 (1:100; Proteintech), anti-CD166 (1:100; Proteintech), anti-CD3 (1:100; R&D systems, USA), anti-Thy1 (1:100; Abcam), anti-Fn1 (1:100; Abcam), anti-EMCN (1:100; Abcam), anti-CGRP (1:100; Cell Signaling Technology, USA), anti-Dcn (1:100; Abcam), anti-CD86 (1:100; Abcam), and anti-CD206 (1:100; Abcam). Next, the sections were incubated with fluorescence-conjugated secondary antibodies (Jackson ImmunoResearch, USA). DAPI was used for nuclear staining. Three distinct subregions in each section were randomly captured with a light/fluorescence microscope (BX53, Olympus). Quantitative analyses were performed in a blinded fashion with ImageJ software.

**Western blot**

Total proteins from tissue homogenates were harvested by RIPA Buffer (Solarbio) supplemented with phenylmethylsulfonyl fluoride, phosphatase and protease inhibitor (Sigma-Aldrich, USA). The concentration of total proteins was measured by a bicinchoninic acid (BCA) protein assay kit (Thermo Fisher Scientific). The aliquot of proteins was separated by 10% SDS-PAGE gel, transferred electrophoretically to polyvinylidene fluoride membranes (Millipore, USA), and blocked in 5% non-fat milk. The membranes were incubated with primary antibodies including anti-COL2A1 (1:1000; Novus), anti-Fn1 (1:1000; Abcam), *p*-Fak (1:1000; Cell Signaling Technology), Fak (1:1000; Cell Signaling Technology), *p*-Akt (1:1000; Cell Signaling Technology), Akt (1:1000; Cell Signaling Technology), and anti-β-actin (1:10000; Proteintech). After washing, the blots were probed with HRP-conjugated secondary antibodies (1:10000; Cell Signaling Technology) and subjected to enhanced chemiluminescence detection (Thermo Fisher Scientific). The intensity of bands was quantified by ImageJ and normalized to the density of the internal control. The uncropped gel images were shown in fig. S11.

**Fig. S1. MagPie exerts good biocompatibility in vitro.**

(**A**) Live/Dead staining of chondrocytes cultured under the following conditions: blank, RPLLA film, and MagPie. Scale bar: 200 μm. (**B**) CCK-8 assay of chondrocytes cultured under the following conditions: blank, RPLLA film, and MagPie. Data were presented as means and SEM. *n* = 3 per group.

**Fig. S2. Food intake of rats from different groups**.

**Fig. S3. Effects of MagPie on subchondral bone improvement in an ADD-induced TMJOA rat model.**

(**A**) Micro-CT images of TMJ condyles 4 weeks post-operation: sham surgery, ADD surgery, ADD surgery with nonpiezoelectric RPLLA film implantation, and ADD surgery with piezoelectric MagPie implantation. The yellow arrows indicate condylar surface flattening and local sclerosis. Scale bar: 1 mm. (**B, C**) Quantitative analysis of height of TMJ condylar heads (B) and BMD in subchondral bone (C) determined by micro-CT measurements. Data were presented as means and SEM. *n* = 6 per group.

**Fig. S4. Dot plot showing the expression of marker genes in cells from cartilage tissue.**

(**A**) Dot plot showing the expression of representative marker genes for different cell types in each cluster. (**B**) Dot plot showing the expression of representative marker genes for myeloid cells in each cluster.

**Fig. S5. Enriched GO terms of biological process in chondrocyte clusters.**

**Fig. S6. Prg4^+^ cells exhibited progenitor-like properties in vivo.**

(**A**) Immunofluorescence staining of Prg4 (red) and EdU (green) in a LRC experiment after 8 weeks. The white arrows indicate Prg4^+^/EdU^+^ cells in superficial zone of cartilage. (**B**) Quantification of results from (A). Data were presented as means and SEM. *n* = 4 per group. (**C**) Representative images quantitative analysis of Prg4 (red) and CD105 (green) double positive cells in TMJ sagittal sections. The white arrows indicate positive cells, and the white asterisks indicate negative signals in cartilage. Scale bar: 100 μm. Data were presented as means and SEM. *n* = 3 per group. (**D**) Representative images quantitative analysis of Prg4 (red) and CD166 (green) double positive cells in TMJ sagittal sections. The white arrows indicate positive cells in cartilage, and the white asterisks indicate negative signals. Scale bar: 100 μm. Data were presented as means and SEM. *n* = 3 per group. (**E**) Representative images quantitative analysis of Prg4 (red) and CD3 (green) double positive cells in TMJ sagittal sections. The white asterisks indicate negative signals in cartilage. Scale bar: 100 μm. Data were presented as means and SEM. *n* = 3 per group.

**Fig. S7. MagPie promoted chondrocytes to secrete Prg4 in vitro.**

(**A**) Immunofluorescence staining of Prg4 (red) and nuclei (blue, stained with DAPI) deposited by chondrocytes cultured under the following conditions: no pressure, cyclic pressure alone, cyclic pressure on RPLLA film, cyclic pressure with Mg^2+^ treatment, and cyclic pressure on MagPie film. Scale bar: 50 μm. (**B**) Quantitative analysis of the percentage of Prg4 positive chondrocytes. Data were presented as means and SEM. *n* = 3 per group. (**C**) Relative expression of the chondrogenic gene *Prg4*. Data were presented as means and SEM. *n* = 5 per group.

**Fig. S8. Heatmap of cell type-specific binarized regulon activity.**

Rows represent regulon activity (binarized to “on” = black or “off” = white).

**Fig. S9. Regulon specificity scores in different chondrocyte clusters.**

The top 5 ranked Regulons are labeled in the plot. Higher RSS values indicate stronger potential associations with cell-type specificity.

**Fig. S10. Schematic illustration of the KEGG pathway.**

**Fig. S11. Raw data of Western blot related to Fig. 7K.**

**Table S1**

**Primer pairs used in RT-qPCR analysis**

| Gene | Forward Primer (5’-3’) | Reverse Primer (5’-3’) |
| --- | --- | --- |
| *Prg4* | CAGGCAGCACAGGTCAGGAG | CTTTGATGGGCACCGAGTAATGG |
| *Gapdh* | AAGTTCAACGGCACAGTCAAGG | TCCACGACATACTCAGCACCAG |
